# The Influence of Essential Oils on Gut Microbial Profiles in Pigs

**DOI:** 10.3390/ani10101734

**Published:** 2020-09-24

**Authors:** Modestas Ruzauskas, Elena Bartkiene, Arunas Stankevicius, Jurga Bernatoniene, Daiva Zadeike, Vita Lele, Vytaute Starkute, Paulina Zavistanaviciute, Juozas Grigas, Egle Zokaityte, Arnoldas Pautienius, Grazina Juodeikiene, Valdas Jakstas

**Affiliations:** 1 Microbiology and Virology Institute, Lithuanian University of Health Sciences, Tilžes g. 18, LT-47181 Kaunas, Lithuania; 2 Department of Anatomy and Physiology, Immunology Laboratory, Lithuanian University of Health Sciences, Tilzes g. 18, LT-47181 Kaunas, Lithuania; arunas.stankevicius@lsmuni.lt (A.S.); juozas.grigas@lsmuni.lt (J.G.); arnoldas.pautienius@lsmuni.lt (A.P.); 3 Institute of Animal Rearing Technologies, Lithuanian University of Health Sciences, Tilzes g. 18, LT-47181 Kaunas, Lithuania; elena.bartkiene@lsmuni.lt (E.B.); vita.lele@lsmuni.lt (V.L.); vytaute.starkute@lsmuni.lt (V.S.); paulina.zavistanaviciute@lsmuni.lt (P.Z.); egle.zokaityte@lsmuni.lt (E.Z.); 4 Department of Food Safety and Quality, Lithuanian University of Health Sciences, Tilzes g. 18, LT-47181 Kaunas, Lithuania; 5 Institute of Pharmaceutical Technologies, Medical Academy, Lithuanian University of Health Sciences, Sukileliu pr. 13, LT-50161 Kaunas, Lithuania; jurga.bernatoniene@lsmuni.lt (J.B.); valdas.jakstas@lsmuni.lt (V.J.); 6 Department of Drug Technology and Social Pharmacy, Lithuanian University of Health Sciences, Eivenių str. 4, LT-50161 Kaunas, Lithuania; 7 Department of Food Science and Technology, Kaunas University of Technology, Radvilenu Rd. 19, LT-50254 Kaunas, Lithuania; daiva.zadeike@ktu.lt (D.Z.); grazina.juodeikiene@ktu.lt (G.J.)

**Keywords:** *Origanum vulgare*, *Mentha piperita*, *Thymus vulgaris*, pigs, probiotic bacteria, microbiota

## Abstract

**Simple Summary:**

In recent years, the intake of ultra-processed foods has increased dramatically worldwide. Missing natural foods in the diet raise the need of biologically active food components that could compensate for this deficiency and help maintain proper immune status. In this study, the microbial changes in pigs as experimental animals were assessed as influenced by consumption of oregano extract combination with peppermint and thyme essential oils. The results demonstrated that the combination of plant extracts had a positive effect on the gastrointestinal tract of animals by increasing the number of probiotic bacteria. Based on the results obtained it may be outlined that the combination of oregano extract and peppermint and thyme essential oils can be promising ingredient as a functional component for the development of the new nutraceutical preparation.

**Abstract:**

In recent years, the intake of ultra-processed foods has increased dramatically worldwide. Missing natural foods in the diet raise the need of biologically active food components that could compensate for this deficiency and help maintain proper immune status. This study used pigs as an animal model for the assessment of the impact of consumption of *Origanum vulgare* plant extract combined with *Mentha piperita* and *Thymus vulgaris* essential oils on microbial profile in intestines. A single group of weaned pigs received basal diet, while the other group basal diet supplemented with plant extract and two essential oils in the form of bilayer tablets prepared using “liquid/solid” phase technology. Metagenomic sequencing was performed with the aim to investigate changes of microbial communities in ileum, caecum, and colon. The results demonstrated that the combination of essential oils was non cytotoxic, and had a positive effect on the microbial composition in the large intestine of pigs due to significant increase in the number of probiotic bacteria. The amount of *Lactobacillus* was 2.5 times and *Bifidobacterium* 1.9 times higher in the animal group fed with supplement. The combination, however, had some negative impact on the variety of minor species in the distal part of the ileum. Additional studies need to be performed to obtain knowledge on how combinations of essential oils can change bacterial variety in the proximal part of the gastrointestinal tract.

## 1. Introduction

Research over the past years has shown that appropriate nutrition is a very important factor for ensuring the good immune status and enhancing the resistance to infections [1]. However, it is not always possible to achieve good nutritional status via the diet alone. In the modern diet, even in industrialized nations, the amount of food has increased, but the quality has decreased due to processing, and it adversely affects an individual’s micronutrient status. The increased consumption of ultra-processed foods requires exploration of natural ingredients to include in the diet. Therefore, there is a great deal of interest in research on specific nutrition interventions that could enhance immune function in sub-clinical situations and so prevent the onset of infections [2]. The problem of antimicrobial resistance must also be addressed by looking for biologically active substances that can become alternative to antibiotics. Some biologically active substances act on pathogenic bacteria while the others are known as modulators of immune system. Various biologically active substances are already known as modulators of immune system. Such compounds include polyphenols [3], micronutrients, and vitamins [4]. Different ingredients of functional foods have been investigated previously. Those include functional enzymes, probiotics, prebiotics, fibres, phytosterols, peptides, isoflavones, saponins, and others. Most of experiments have been performed in vitro; however, the data might be different in live organisms, as multiple factors may influence the activity of these substances. This is especially important when selecting a few or multiple ingredients in a single formulation. Recently, we have investigated some functional products for the active nutraceutical formula which could benefit both humans and animals. Some constituents including bovine colostrum, probiotics and particularly essential oils (EOs), demonstrated good results in vitro [5,6,7,8]. Essential oils are complex mixtures of volatile compounds isolated from a whole plant or plant part of known taxonomic origin [9]. EOs are generally considered natural and less toxic compounds in comparing with other natural products such as antibiotics [10]. EOs act against wide spectrum of pathogenic bacteria at relatively small concentrations and can be used in combination with other active ingredients, such as bovine colostrum or lactic acid bacteria, in an appropriate technological formula [5]. Moreover, EOs have anti-inflammatory potential and immunomodulatory effect [11,12].

By above-mentioned reasons, EOs are promising substances for the treatment and prophylaxis of infectious diseases both in humans and animals. Recent experiments demonstrated that EOs are active against wide spectrum of antimicrobial resistant isolates including methicillin-resistant *Staphylococcus aureus*, extended-spectrum beta-lactamases, and carbapenemases-producing *Enterobacteriaceae* isolated from humans and animals [5,13]. Moreover, EOs have shown promise as antiviral agents against several pathogenic viruses [14]. Different studies in animals and humans suggest that EOs have immune function enhancing or immune function balancing properties [15]. EOs have been successfully applied as feed additives to broiler chickens or weaned piglets and have clearly shown immunostimulatory effects [16]. EOs are rich sources of natural antioxidants, such as the phenolic compounds and due their high redox properties and chemical structure have the ability to neutralize free radicals, chelate transitional metals, and quench singlet and triplet oxygen by delocalization or decomposition of peroxides both in humans and animals [17,18,19]. These properties of EOs can benefit both human and animal health.

Up to date, multiple experiments have been performed on EOs action on bacteria in vitro, although there is a lack of knowledge on how EOs influence microbial communities in vivo under natural conditions. Recent advances in Next Generation Sequencing allow us to explore microbial communities very efficiently, compared with investigations based on classical microbiological methods. As there is still a lack of information how do EOs acts on the human metabolism and microbial composition, many studies have been performed using animals and particularly pigs as experimental model. The pig model is one of the best preclinical animal models for investigating the gastrointestinal system and its functions in humans and it is widely used for in vivo experiments searching for healthier foods [20,21]. The possibility to feed animals with supplementation of EOs is also promising from few perspectives including economic reasons and reducing of antimicrobial usage. There are data that the cinnamaldehyde and thymol (250 mg/kg) can significantly improve digestibility of dry matter, crude protein, and energy in piglets [22,23]. The apparent ileal digestibility of crude protein and most amino acids were improved by the cocktail of EOs (300 mg/kg) with menthol as the primary component [24]. The mechanism of action of EOs is not very clear, but according to some studies, the improved digestibility can be attributed to the enhanced secretion of bile and enzymes [25] and to the decreased endogenous losses of nutrients [24]. EOs can also modify intestinal microbial composition in weaned piglets and therefore can enrich biosynthesis of proteins and to activate metabolism of lipids [26]. EOs have also been shown to regulate intestinal contraction, and thereby to influence the transit of digesta and the resultant interaction between feed and the endogenous enzymes. In pigs the active substances of EOs were found to be absorbed nearly completely in the stomach and the proximal small intestine within 2 h after oral administration [27]. This means that the absorption of EOs in the foregut protects the possibility of active substances to reach large intestines and directly act on the microbial communities. It is known that microbial changes in the large intestines appear after supplementing of feed by unprotected EOs [26]. This means that there are other pathways to active substances of EOs interacts with gut microbial communities. However, for the different drug relaxation in the gastrointestinal tract (GIT) different types of pharmaceutical coatings are used. The “liquid/solid” coating technique is capable addition toward such an aim for solubility enhancement and dissolution improvement, thereby increasing the bioavailability. It contains liquid medications in powdered form. This technique is an efficient method for formulating water insoluble and water soluble drugs [28]. 

It is known that EOs can act on the gut microbiota selectively. For instance, a cocktail of carvacrol, cinnamaldehyde, and capsicum oleoresin increased the population of *Lactobacilli* and the ratio of *Lactobacilli* to *Enterobacteriaceae* in the jejunum and cecum of early-weaned piglets [29]. The way EOs active substances can modify microbial composition is very important as it is known that the microbiota plays a fundamental role in the induction, training, and functioning of the host immune system [30]. It also actively participates in the digestion process. Bacteria belonging to phylum Firmicutes are able to digest long-chain carbohydrates and thus provide the host with additional sources of nutrients [31]. In addition to its positive role, microbiota has a potential in the development of irritable bowel disease and some other diseases, as well as obesity [32]. It may also elaborate toxic products from food residues such as genotoxic hydrogen sulphide [33]. As the microbiota has the potential to play different roles in the gut of the host, it is very important to explore the key elements influencing the domination of beneficial bacteria over the pathogenic ones or those that may have a negative impact on health. Different EOs have been investigated regarding their antimicrobial and functional properties. The previous investigations proved that the potential of activity of different EOs is not equal [34]; therefore, the selection of an appropriate EOs with the aim to obtain the most active (or optimal) composition is very important.

Oregano (*Origanum vulgare*) is a worldwide herb that is extensively used due to its medicinal activities against respiratory, digestive, and urinary disorders in addition to dental caries and rheumatoid arthritis [35]. This plant also has strong antioxidative properties [36]. The aforementioned pharmacological properties of *Origanum vulgare* are attributed to its high content in carvacrol and other phenolic compounds [37]. Peppermint (*Mentha piperita*) is another widespread plant recognized for their special qualities such as anti-bacterial, stimulative, diaphoretic, stomachic, and anti-spasmodic. It reliefs against cold, flu, fever, anorexia, nausea, motion sickness, food poisoning, esophagus and sinus illnesses [38,39,40]. Thyme (*Thymus vulgaris*), which belongs to the Lamiaceae family, is rich source of phytochemicals and bioactive compounds. [41]. Thyme EO also inhibits growth of pathogenic bacteria and is known as an anti-fungal agent [42]. Among this, thymus possesses antispasmodic, diaphoretic, antiseptic, disinfectant, carminative, sedative, and expectorant properties that are due to thymol and carvacrol [43]. As aforementioned, herbs have wide spectrum of activity the composition of herbs and their EOs are expected to have even wider spectrum of activity including their ability to act on microorganisms and therefore to modify microbiota within the gut. The possibility to reduce the dose of a single essential oil and the usage of a cocktail of EOs can be important to reduce the toxicity of a single active substance as the main active substances from EOs such as thymol and carvacrol are known as toxic at least for plants and insects [44,45]. The necessity to explore EOs influence on the microbial communities in the gut is actual for both humans and animals. With the aim to develop nutraceuticals we have explored different active ingredients including bovine colostrum, probiotics and various plant by-products. Here we have investigated the mix of EOs with possibility to include the selected composition as ingredient into the nutraceutical formulation. 

The aim of this study was to evaluate the cytotoxicity of *Origanum vulgare* plant extract, *Mentha piperita*, and *Thymus vulgaris* EOs and to evaluate microbial changes in the guts of pigs as experimental model by supplementing their diet with a combination of the aforementioned plant extract and two essential oils (EEOs) given in the form of bilayer coating tablets.

## 2. Materials and Methods 

### 2.1. Plant Material

Dried *Origanum vulgare* herb was obtained from “Zolynu namai” (Vilnius, Lithuania). Voucher specimens (No. L170712) were deposited at the Herbarium of the Department of Drug Technology and Social Pharmacy, Lithuanian University of Health Sciences, Lithuania. Oregano herb was ground in a cross beater mill IKA A11 Basic Grinder (IKA Works, Guangzhou, China) and sieved using a vibratory sieve shaker AS 200 basic (Retch, United Kingdom) equipped with mint (*Mentha piperita*) essential oil and thyme (*Thymus vulgaris*) essential oil purchased from Sigma-Aldrich (St. Louis, MO, USA).

### 2.2. Preparation of Oregano Herb Extract 

Dried powdered plant material (100 g) has been extracted with 1000 mL of 70% ethanol + 30% glycerol (purity 98%) in a round bottom flask by heat-reflux extraction performed in a water bath for 4 h at 95 °C. The extract has been passed through a vacuum filter.

### 2.3. Formulation of “Liquid/Solid” Phase with Essential Oils

The innovative technology in the “liquid/solid” phase of the system has been used to improve the bioavailability of sparingly water-soluble active compounds (thyme and mint essential oils) and has been used for powder production. In the “liquid/solid” phase of the system, the technology was carried out: using a simple mixing method, where the solution of active ingredients and excipients were mixed until the formation of suitable powder properties. Magnesium aluminum metasilicate was chosen as the carrier for powder production in the “liquid/solid” phase. The carrier and the mixture of ethanolic oregano extract and mint and thyme essential oils were mixed together in a mortar, sieved through a 1 mm sieve and homogenized for 10 min in a mixer. The prepared mixture was placed in an oven at 60 °C for 1 h, the procedure being repeated until all the solution has been used. The prepared mixture was placed in a ventilated dryer at 40 °C for 24 h. After drying, a free-flowing, yellowish powder was obtained. Chromatographic analysis showed that the amounts of the main biologically active compounds, thymol, carvacrol and menthol, were not significantly reduced in the powder formulations. The liquid/solid powder was further used in the tableting process. The resulting bulk and high-quality powder was tableted into mini-tablets, which are coated with a bilayer coating for different relaxation in the GIT. 

### 2.4. EEOs Combination

The EEOs combination was prepared from the oregano (*Origanum vulgare*) extract, which contained at least 13.2 g/L of carvacrol, peppermint (*Mentha piperita*) essential oil, which contained at least 1 g/L of menthol, and thyme (*Thymus vulgaris*) essential oil, which contained at least 4 g/L of thymol and 0.1 g/L of carvacrol. The ratio of oils in the prepared combination was 99:0.3:0.7, respectively. The suspension obtained was used for tablet production with Neusilin (Fuji Chemical Industries, Toyama, Japan) as adsorbent. 332 mg tablets were produced using the single punch eccentric tablet press Erweka RTP-D8 (Langen, Germany). The tablets were coated with an Erweka AR 403 apparatus with a drilling rig D63150. Coated with a 10% shellac solution containing 2.8 oleic acid and 10 mL of a 10% ammonia solution in a 43% ethanol solution. The weight of the coating is 5% of the weight of the tablet. 

The tablets were manufactured using an Erweka RTP-D8 eccentric press. Press speed 4 pcs, compression force 5N. Tablet weight 332 mg. The obtained results are shown in Table 1.

### 2.5. Cytotoxicity Testing of EEOs

The following cell lines were used: pig kidney cells (PK-15 ATCC No. CCL-33) and monkey kidney cells (MARC-145 ATCC No. CRL-12231; Vero ATCC No. CCL-81). All cell lines were cultured in Minimum Essential Medium (MEM; Gibco) with additional 10% heat-inactivated fetal bovine serum (FBS; Gibco), 100 U mL^−1^ penicillin and 100 μg mL^−1^ streptomycin. Cells were grown at 37 °C in humidified 5% CO_2_ and 95% air mixture. Monolayers of cells were trypsinized, diluted (1:6 dilutions for PK-15 cells and 1:4 for MARC-145 and Vero cell lines, respectively) in growth medium and transferred to 25 cm^2^ tissue culture flasks (TPP Techno Plastic Products AG, Trasadingen, Switzerland). The extract of oregano and the mint essential oil and thyme essential oil were evaluated on pig kidney cells (PK-15) and monkey kidney cells (MARC-145, Vero) in order to examine their cytotoxic effects against normal cells. The cells were seeded on 96-well plates (1× 10^4^ cells/well) and incubated at 37 °C with 5% CO_2_. After 48 h incubation, the cells were treated with the extracts (from 1 μL/mL to 50 μL/mL) for 24 h and 48 h at 37 °C. The essential oil extracts were dissolved in MEM. Following the removal of the extracts from the wells, the cells were washed in phosphate buffered saline. Cell viability was determined by MTT assay. The cytotoxicity of essential oil extracts was estimated against PK-15, Marc-145 and Vero cells using MTT assay [46]. Each extract was tested in triplicate. After 72 h MTT reagent (10 μL, 5 mg/mL, Sigma-Aldrich) was added and incubated for 4 h at 37  °C. Then, 100 μL dimethyl sulphoxide (DMSO) (Carl Roth, Germany) was added into each well, and the plates were placed on the shaker for 5 min. The absorbance of each well was measured at 620 nm in a microplate reader (Multiskan™ FC Microplate Photometer, Thermo Fisher Scientific Oy, Ratastie, Finland), and the percentage of cell survival was calculated. Viability was defined as the ratio (expressed as a percentage) of absorbance of treated cells to that of untreated control cells.

### 2.6. Chemical Analysis of Essential Oils and Tablets

The composition analysis of essential oils and tablets was performed using a Shimadzu GC-2010 gas chromatograph (Shimadzu, Tokyo, Japan) equipped with a Shimadzu autoinjector AOC-20is (Shimadzu, Tokyo, Japan). The operational conditions for essential oils analysis (thyme and mint) were as follows: temperature program from 0 °C to 50 °C (2 min), from 50 °C to 215 °C (2 min) at 2 °C/min, and then from 215 °C increased to 310 °C (6 min) at 40 °C/min, pressure 69.6 kPa, total flow 65.2 mL/min, column flow 1.22 mL/min, and linear velocity 40 cm/sec. A column RXI-5MS (30 m × 0.25 mm i.d. × 0.25 μm film thickness) was used. Split injector temperature: 240 °C. Split ratio: 1:50. The operational conditions for tablets analysis were as follows: temperature program from 0 °C to 70 °C (2 min), from 70 °C to 250 °C (2 min) at 20 °C/min, then from 250 °C increased to 300 °C (3 min) at 50 °C/min, pressure 76.4 kPa, total flow 16.1 mL/min, column flow 1.19 mL/min, and linear velocity 40 cm/sec. Split injector temperature: 290 °C. Split ratio: 1:10. 

### 2.7. In Vivo Experiment Design

In vivo experiment was performed using 16 weaned-off 17–19 kg Lithuanian White breed 56-day-old pigs in September 2019. Pigs were bred and delivered from the large capacity pig breeding farm to the Biological Research Centre of Lithuanian University of Health Sciences keeping requirements of animal welfare. Animals were selected from the same two related litters, with the aim to obtain individuals with more similar physiological parameters including body weight. All animal procedures were conducted according to the EU Directive of the European Parliament and of Council from 22 September 2010 [47] on the protection of animals used for scientific purposes and Requirements for the Keeping, Maintenance, and Use of Animals Intended for Science and Education Purposes (2010/63/EU), approved by the order of the Director of the Lithuanian State Food and Veterinary Service [48]. The number of permission for this study is G2-123. The animals were divided into two groups (control group and experimental group), eight pigs (four males and four females) in each group in separate pens within the same room. All animals were fed regular animal feed, which was prepared according to the standard recipes. Regular feed consisted of barley (44.6%), wheat (20%), soybean meal (7.9%), whey powder (6.5%), corn (5%), soybean protein (3%), sugar beet pulp (3%), and potato protein (2%). Before experiment, the animals were kept in pens for 5 days for adaptation. All animals in the experimental group additionally received ~1 g of the preparation (3 tablets) daily, during the morning feeding until the end of the experiment. This corresponds to 986 mg of oregano extract, 3 mg of peppermint essential oil and 7 mg thyme essential oil. The preparation was added to the feed ensuring that it was consumed by each animal. Duration of the experiment was 30 days. Twenty-four hours before the experiment, fecal content from the rectum was taken for microbiome studies for the comparison of microbial variety among the groups. On the 30th day, the experiment was finished, and animals were sedated and euthanized according to the requirements laid down in the Directive.2010/63/EU. The content (5 g) from the distal part of the ileum, caecum and the central part of the colon was taken into sterile DNase and RNase free tubes, delivered to the Institute of Microbiology and Virology, where specimens were preserved using DNA/RNA Shield (Zymo Research, Irvine, USA) and stored at −20 °C. The material was used for microbiome studies in experimental and control animal groups.

### 2.8. Metagenomics and Microbial Profiling

The DNA from each sample was purified using a fecal DNA MiniPrep kit (D6010, Zymo Research, Irvine, CA, USA) according to manufacturer’s instructions. The obtained DNA then was purified using a DNA Clean & Concentrator-25 kit (Zymo Research, Irvine, CA, USA) to produce about 50 ng/µL of DNA. The same amount of DNA from each group of animals was pooled into one sample per group representing one sample per intestine i.e., three samples were obtained from each group representing samples from ileum, caecum and colon. In total, six samples were obtained representing three intestines from both animal groups. Partial 16S rRNA genes (V3-V4) were PCR amplified followed by barcoding and library preparation of the resulting PCR products in a secondary PCR. Metagenomic libraries were prepared, sequenced, quality controlled, and assembled in an independent service laboratory (BaseClear, The Netherlands). Short paired sequence reads were generated using the Illumina MiSeq system (Illumina, IL, USA) and converted into FASTQ files using the BCL2FASTQ pipeline software, version 1.8.3 (Illumina, IL, USA). Subsequently, reads containing PhiX control signal were removed using an in-house filtering protocol. In addition, reads containing (partial) adapters were clipped (up to a minimum read length of 50 bp). The second quality assessment was based on the remaining reads using the FASTQC quality control tool version 0.11.5. Subsequently, the Illumina paired reads were merged into single reads (so-called “pseudoreads”) through sequence overlap. Chimeric pseudoreads were removed, and the remaining reads were aligned to a combination of the GreenGenes and RDP 16S gene databases. Based on the alignment scores of the pseudoreads, the taxonomic classes were assigned by associating each pseudoread to the best matching operational taxonomic unit (OTU). The results of the taxonomic classification were presented on the interactive online platform. The number of probiotic bacteria was recorded and the relative number of probiotics from all bacteria detected was counted in all intestines tested as an average number per group. The bacterial genera considered as probiotic bacteria was based on literature analysis [49,50,51] and included *Lactobacillus*, *Bifidobacterium*, *Bacillus*, *Faecalibacterium*, *Streptococcus*, *Enterococcus*, and *Escherichia*. Moreover, all the bacterial genera detected in the amount of ≥2%, irrespective of the animal group, were checked as to whether they were not mentioned as probiotic bacteria in scientific manuscripts.

### 2.9. Statistical Analysis

Differences of microbial profile between the groups were assessed using Z score calculator for two population proportions (https://www.socscistatistics.com/) [52] evaluating the proportion of total number of OTU and OTU of separate taxonomic units prevalence, all of which was compared in the experimental and control animal groups. The Kruskal–Wallis test was used for multigroup comparisons of tested cytotoxicity. The results were considered statistically significant at *p* < 0.05.

## 3. Results

### 3.1. Chemical Analysis of EOs

**Figure 1:**
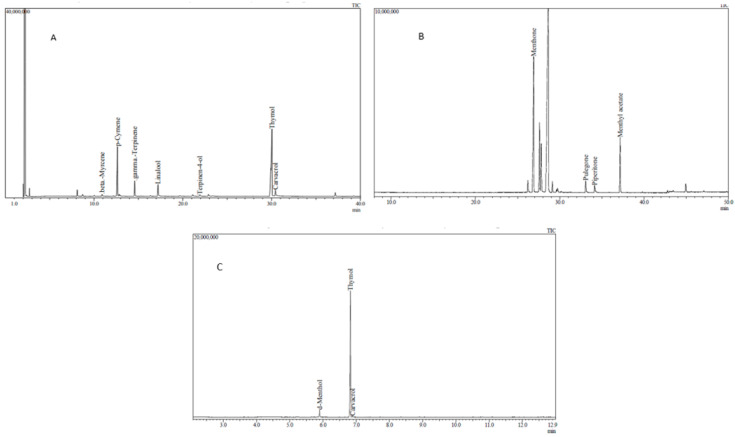
Gas chromatography-mass spectrometry chromatograms: (**A**) thyme essential oil; (**B**) peppermint essential oil and (**C**) tablets with *Origanum vulgare* plant extract, *Mentha piperita*, and *Thymus vulgaris* essential oils.

The volatile compounds obtained from thyme essential oil were beta-myrcene, p-cymene, gamma-terpinene, linalool, terpinen-4-ol, thymol, and carvacrol (Figure 1A). Thymol was the predominant compound of the thyme essential oil composition (59.71%). The volatile compounds obtained from mint essential oil were menthone, pulegone, piperitone, and menthyl acetate (Figure 1B). The predominant compound in this composition was menthone (67.13%). According to the results of the gas chromatography-mass spectrometry (GC-MS) analysis the main compounds in the tablets were obtained. The predominant compounds of the tablets were d-menthol, thymol, and carvacrol (Figure 1C). 

### 3.2. Cytotoxic Effects of EEOs

**Figure 2:**
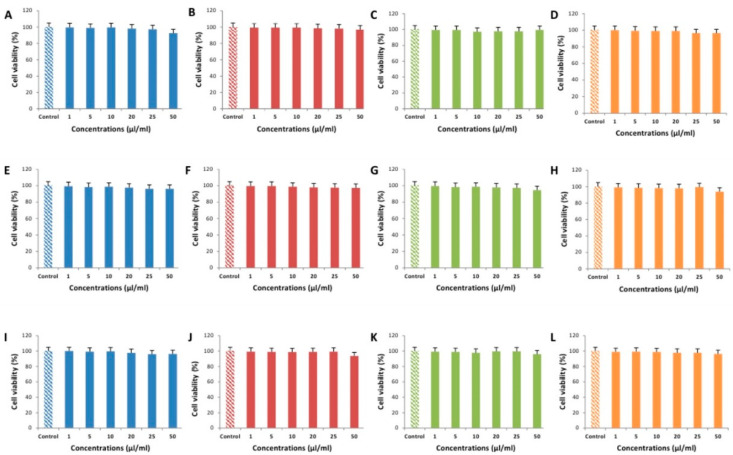
Effect observed in MARC-145 (**A**–**D**), PK-15 (**E**–**H**), and Vero (**I**–**L**) cell lines, caused by oregano extract (carvacrol) (**A**,**E**,**I**; colored blue), mint essential oil (menthol) (**B**,**F**,**J**; colored red), thyme essential oil (**C**,**G**,**K**; colored green), and a mixture of all tested compounds (**D**,**H**,**L**; colored orange) in concentrations of 1, 5, 10, 20, 25, and 50 µl/mL concentrations. Each bar represents mean ± SD of triplicate sample data. Concentration multigroup comparisons were performed using Kruskal-Wallis test.

The results of cell cultures viability tests obtained using different bioactive compounds and their mixtures, demonstrated that the oregano extract, mint essential oil, thyme essential oil, and the mixtures of all the tested compounds in concentrations of 1–50 µL/mL did not show any cytotoxic effect after 48 h in MARC-145, PK-15 and Vero cell lines. Cytotoxic effects of the extracts on the viability of cell cultures are summarized in Figure 2. 

Cell viability in all tested cell lines did not fall below 99% in the 1 µL/mL exposure group. Except for PK-15 cell line, where the exposure to 5 µL/mL of oregano extract, mint essential oil (Figure 2E), and the mixture of all components (Figure 2H) resulted in cell viability of around 98%, cell viability did not fall below 99% in the 5 µL/mL exposure group. In the 10 µL/mL exposure group, only MARC-145 cells exposed to thyme essential oil showed cell viability lower than 98% (Figure 2C). Interestingly, while cell viability in all tested cell lines did not fall below 97% in the 20 µL/mL exposure group, in the 25 µL/mL exposure group, a 5 µL/mL difference resulted in cell viability falling below 97% in MARC-145 cells exposed to the mixture of all components (Figure 2D), Vero cells exposed to oregano extract (Figure 2I) and PK-15 cells exposed to oregano extract (Figure 2E). While the cell viability of the majority tested cell lines did not fall below 96% at 50 µL/mL exposure concentrations, MARC-145 cells exposed to oregano extract (Figure 2A) reached cell viability of 92.67% (CI95% 97.33–88.01), Vero cells exposed to mint essential oil (Figure 2J) reached cell viability of 93.67% (CI95% 98.33–89.01), while PK-15 cells exposed to thyme essential oil (Figure 2G) and the mixture of all compounds (Figure 2H) reached cell viability of 94.67% (CI95% 99.33–90.01) and 94.00% (CI95% 98.66–89.34), respectively.

### 3.3. Microbial Profiles in the Gut of Experimental Animals

Before experiment, the most prevalent bacterial classes in pig faces were *Bacteroidia* and *Clostridia*, whose general prevalence varied from 69.4% to 72.1% in all microbiota in the experimental and control groups. The other frequently detected classes were *Negativicutes*, *Bacilli*, and *Gammaproteobacteria*. The composition of microbiota at a class level was similar among the groups. 

**Figure 3:**
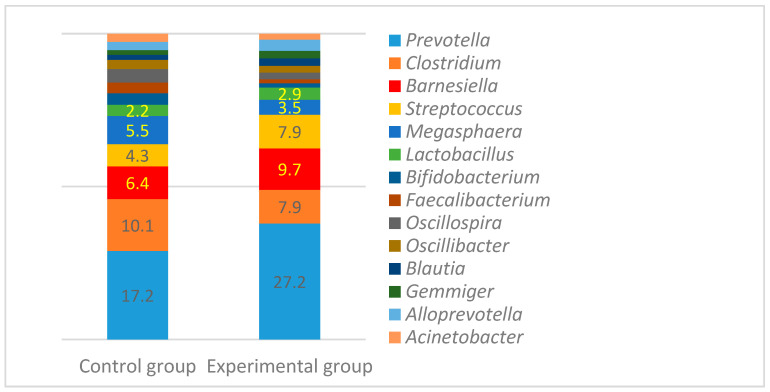
The most prevalent bacterial genera (%) among the control and experimental groups before the experiment.

The most prevalent bacteria at the genus level in both groups of pigs were *Prevotella*, *Clostridium*, *Barnesiella*, *Streptococcus*, and *Megasphaera* (Figure 3). *Lactobacillus* and *Bifidobacterium* were also detected with the prevalence from 3.5% to 5.5% *(Lactobacillus*) and from 2.2% to 2.9% (*Bifodobacterium*). The overall genus variety and the number of predominant bacteria were similar among the groups with the highest difference found in the number of *Prevotella,* which was higher (27.2% vs. 17.2%) in the control animal group (*p* < 0.05). 

**Figure 4:**
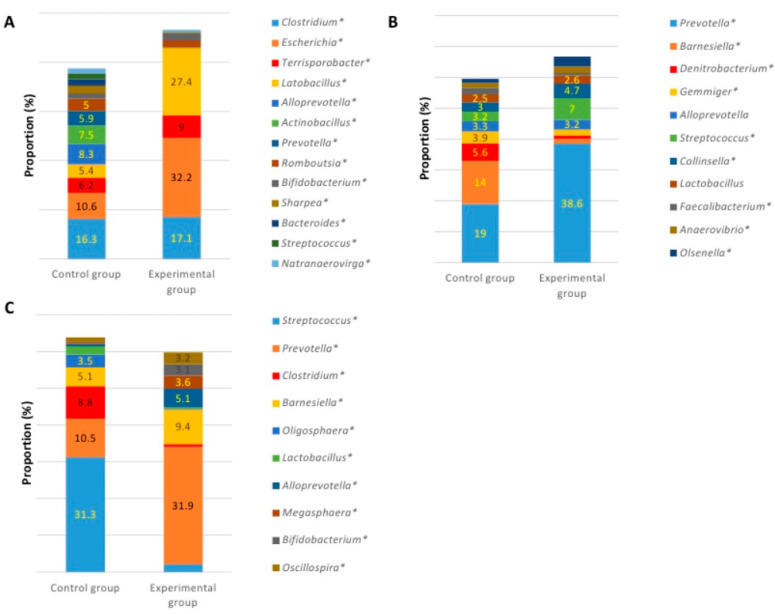
Microbial profiles at the genus level in ileum (**A**), caecum (**B**), and colon (**C**) content after the experiment (genera with the prevalence of ≥2%). * means significant differences between the groups (*p* ≤ 0.05).

At the end of the experiment, the variety of species in large intestines was similar in both groups. The number of species in the caecum in both groups was 300, including unclassified species with the prevalence of at least 0.01% from all bacterial reads. The number of bacterial species was very similar in the colon as well (289 and 297 species in the control and experimental groups, respectively). Statistically significant differences were detected in the terminal ileum, where the number of species was much higher in the control group (220 vs. 125 species) of pigs. Microbial profiles at the genus level in different intestines at the end of the experiment are presented in Figure 4. 

When comparing bacterial composition in the ileum, high differences were observed between the groups. In the control group, the highest amount of bacteria was observed in populations of *Clostridium* (16.3%), *Escherichia* (10.6%), *Alloprevotella* (8.3%), and *Actinobacillus* (7.5%), while the distribution of genera was quite equal (Figure 4A). In the experimental group, the bacterial amount of four genera was much higher, compared with the numbers of microorganisms of other genera detected in the ileum content. In this case, the most prevalent genera were *Escherichia* (32.2%), *Lactobacillus* (27.4%), *Clostridium* (17.1%), and *Terrisporobacter* (9%). The prevalence of those four genera was 85.7% of all bacterial loads. 

Bacterial composition in the caecum content was more similar between the groups, compared with other intestines. *Prevotella*, *Denitrobacterium*, *Barnesiella*, *Gemmiger*, *Alloprevotella*, *Streptococcus*, and *Collinsella* were among the most prevalent genera in both groups. The biggest difference was observed in the amount of *Barnesiella,* which was more than 12 times higher in the control group, while in the experimental group *Prevotella* was the most abundant genus. The other genera with a higher prevalence in the experimental group were *Streptococcus* (7% vs. 3.2%) and *Collinsella* (4.7% vs. 3%), while in the control group, higher numbers were observed in *Denitrobacterium* (5.6% vs. 1%) and *Gemmiger* (3.9% vs. 1.9%) (Figure 4B). There were no statistically significant differences in the number of *Lactobacillus* in the caecum content between the groups (2.5% vs. 2.6%, *p* > 0.05). Significant differences were detected among the predominant genera in the content of colon between the groups (Figure 4C). In the control group, the most prevalent genera were *Streptococcus*, *Prevotella*, *Clostridium* and *Barnesiella,* whose amount was more than a half of all bacterial loads within this part of the GIT. The amount of *Streptococcus* was very high (31.3%), compared with the other genera in this group. In the experimental group, the most prevalent genus was *Prevotella* (31.9%), followed by *Barnesiella* (9.4%) and *Alloprevotella* (5.1%). The highest differences were detected among the numbers of *Streptococcus* (31.3% vs. 2.1%), *Prevotella* (10.5% vs. 31.9%), *Clostridium* (8.8% vs. 0.8%), *Alloprevotella* (0.7% vs. 5.1%), and Oligosphaera (3.5% vs. 0.2%) in the control and experimental groups, respectively. 

**Figure 5:**
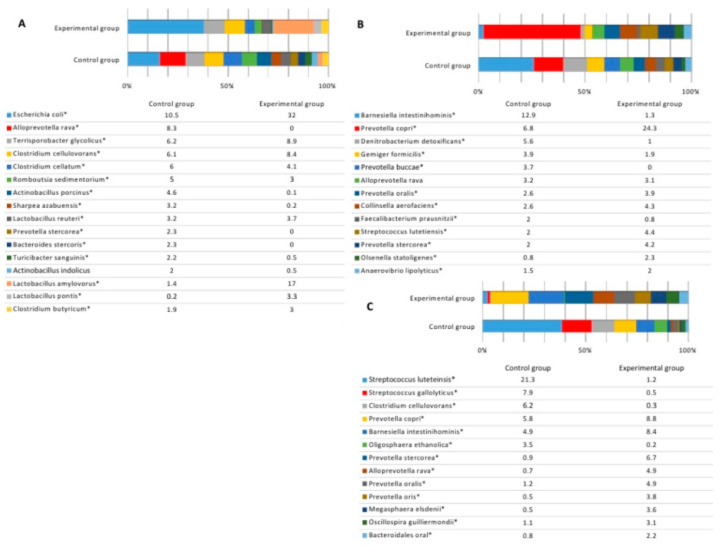
Microbial profiles at the species level in ileum (**A**), caecum (**B**), and colon (**C**) content after the experiment (species with the prevalence of ≥2%). * means significant differences between the groups (*p* ≤ 0.05).

Species distribution among the groups is presented in Figure 5. The data represents the species whose prevalence was at least 2% in any of the animal groups. 

The amount of predominant bacterial species in the ileum of the control group was quite equally distributed (Figure 5A). The highest number of a single species (*Escherichia coli*) in this group was 10.5%, while the numbers of other prevalent species differed only slightly. In the experimental group, on the contrary, there were two species whose numbers were much higher, compared with the rest. The highly predominant species in this group were *Escherichia coli,* with the prevalence of 32%, and *Lactobacillus amylovorus,* with the prevalence of 17%. Despite that, the amounts of separate bacterial species were different between the groups, the general variety of species was quite similar, except for few species (*Alloprevotella rava*, *Prevotella stercorea*, and *Bacteroides stercoris*), which were absent in the experimental group. 

In the caecum, like in the ileum, the bacterial number of separate species changed in a gradually decreasing manner in the control but not experimental group (Figure 5B). The most prevalent species in the groups were the same; however, their numbers differed, particularly regarding the amount of *Prevotella copri,* whose prevalence was 6.8% and 24.3% in the control and experimental groups, respectively. The control group had significantly higher amount of *Barnesiella intestinihominis,* compared with the experimental group, with the prevalence of 12.9% and 1.3%, respectively.

In the colon, the variety of species among the groups was similar, whereas the amount of separate species was unequally distributed. In the control group, the high number of *Streptococcus lutetiensis* (21.3%) was recorded and followed by the other streptococcal species—*S. gallolyticus* (7.9%) (Figure 5C). In the experimental group, the highest prevalence had *Prevotella copri* (8.8%) and *Barnesiella intestinihominis* (8.4%). 

**Figure 6:**
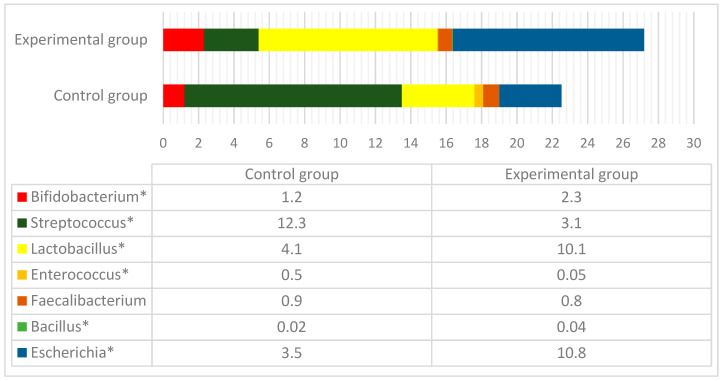
The number (%) of probiotic bacteria in the control and experimental animal groups at the end of the experiment. *- significant differences between the groups.

Figure 6 represents the number of bacterial genera which are considered as probiotic bacteria. 

On average, the number of probiotic bacteria in all three intestines as a part of the total amount of bacterial load was 22.5% and 27.2% in the control and experimental groups, respectively (*p* < 0.05). In the control group, the higher prevalence had *Streptococcus* and *Enterococcus,* while the rest of the genera, including *Lactobacillus* and *Bifidobacterium,* were significantly more abundant in the intestinal content of the experimental group. The only *Faecalibacterium prausnitzii* was equally distributed among the groups.

## 4. Discussion

We studied the influence of the composition of EOs to microbial profiles in different intestines using pig model. Two animal groups used in this experiment came from the same litters, they had similar microbial profiles at the beginning of the experiment. At that period of age, the most prevalent bacterial classes in piglet faces were *Bacteroidia* (phylum Bacteroidetes) and *Clostridia* (phylum Firmicutes), whose prevalence varied from 69.4% to 72.1% depending on the animal group, and this is in coincidence with data obtained by other authors [53,54]. The main genera included *Prevotella*, *Clostridium*, *Barnesiella*, and *Streptococcus,* i.e., normal microbiota usually found in healthy pigs [55].

At the end of the experiment, microbial profiles were examined is separate intestines, as bacteria of different genera unevenly prevail in different parts of the gut [56]. Significant changes were detected regarding bacterial composition in the ileum. Although taxonomical composition was similar in both groups, the amounts of separate genera were unequally distributed in the intestines of experimental group. The main changes were associated with an increase of *Escherichia* (almost three times) and *Lactobacillus* (five times) and a decrease of *Prevotella* (59 times) in the experimental group, compared with control pigs. Both *Escherichia* and *Lactobacillus* are considered as probiotic bacteria [57]; therefore, EOs obviously had a positive effect on the numbers of those genera; however, *Escherichia* is also a possible pathogen and can be unwanted microorganism, depending on its pathogenicity. While virulence factors of *E. coli* were not investigated in this study, it should be taken into account that it could be investigated in forthcoming trials. Although *Prevotella* is usually found in pig GI tract in large amounts [58], there is a lack of information about the importance of this genus in pigs. There are suggestions that *Prevotella* may play important roles to combat post-weaning diarrhoea and that it may contribute to nutrient transport and uptake in the pig gut ecosystem [59]. It is unclear, however, in which part of the GI tract *Prevotella* has the most important role, but according to the study performed previously, this bacterium is mostly distributed in the colon [56]. In our study, we found the highest amount of *Prevotella* present in the caecum, where it was the most predominant genus in both groups of pigs. Moreover, the amount of *Prevotella* in the caecum of experimental animals was significantly higher than in the control group; therefore, the total number of this genus in GI did not decrease in pigs fed with EEOs supplement. The species whose prevalence increased in the ileum of control animals were *Escherichia coli* and *Lactobacillus amylovorus*. The number of *L. amylovorus* increased 12 times and made 17% of total bacterial count from the jejuni. This species has adhesive and significant pathogen inhibitory efficacy and is recognized as potential candidate to be used as probiotic feed supplement [60]. Changes in the caecum content were mostly associated with an increase of *Prevotella* and *Streptococcus* and a decrease of *Barnesiella* and *Denitrobacterium,* compared with the control group. The amount of *Barnesiella,* however, was more than eight times less in the experimental group and was probably replaced by *Prevotella* and *Streptococcus*. Although *Barnesiella* has been found in many other studies investigating pig microbiome, there is still no clear information regarding its functions and significance. *Denitrobacterium* has a single species—*D. detoxificans,* which is better known as ruminant species and its significance in porcine GI is also still under-investigated. The increased genera in the caeca of experimental pigs therefore are considered as positive or even probiotic (*Streptococcus*) microbiota. The most prevalent species in the caeca of the experimental group of pigs was *Prevotella copri,* whose prevalence was 24.3%, and that is 3.5 times higher than in the control group. The role of this species in pigs is not known yet, but *P. copri* is a common human gut microorganism that is both positively and negatively associated with host health [61]. The role of *P. copri* in human health is debatable [62]. In a recent study, De Filippis et al. have detected distinct strains of *P. copri* by metagenome studies and showed that the diet might select distinctive *P. copri* populations [62], but there is still no data about this in pigs. The most prevalent bacterial species in the caeca in the control group was *Barnesiella intestinihominis,* followed by *P. copri*. *B. intestinihominis* was also more prevalent in the colon content of experimental animals; therefore, there were no big differences in this species between the groups. *B. intestinihominis* has been better described in humans than in pigs and has also been mentioned in a study were this species together with *Enterococcus hirae* had a positive effect on the efficacy of cyclophosphamide used for cancer treatment in mice. The prevalence in healthy pigs of species that were previously associated with human microbiota, demonstrates the similarity between human and porcine microbiota. This fact is important as pig model can gain more knowledge about the pathogenesis of different human diseases. Variety of the rest of the species in the caecum content was similar in both groups demonstrating that EEOs did not have a selective inhibitory effect on the main species normally inhabiting the GI of pigs. Such variety similarities were also observed in other large intestines. The amount of some genera and species, however, differed significantly. In the colon, these differences were also noticeable: while in the control group the most prevalent genus was *Streptococcus*, in the experimental group predominated *Prevotella*. Among streptococci the most prevalent species were *S. lutetiensis* and *S. gallolyticus,* whose prevalence reached almost 30%. Those species were also detected in the experimental group but only in a small amount (1.7%). It is not clear yet if these species of streptococci are widely distributed in pigs, but they are frequently encountered in blood cultures of humans with bacteremia, sepsis, and endocarditis [63]. Recently, Piva et al. described the first case of isolation of *S. lutetiensis* from a diseased cat [64]. *S. lutetiensis* and *S. gallolyticus* belong to the group of *S. bovis* from which they were separated into separate species [63]. While *S. lutetiensis* is well known as pathogenic species in humans, particularly in patients with reduced immunity, *S. gallolyticus* is recognized as an inhabitant of the alimentary tract of cattle, sheep, and other ruminants [65]. This study revealed that both *Streptococcus* species can be abundant in the intestinal tract of pigs and proves that pigs are carriers of streptococcal species pathogenic to humans. For this reason, *S. gallolyticus* and, particularly, *S. lutetiensis* can be considered as zoonotic bacteria. On the other hand, this is not certain for types or strains that might differ between the hosts, thus more studies in this direction should be performed. *S*. *gallolyticus* is known to be transmitted either directly or indirectly between animals and humans [66]. It is also important to note that *S. gallolyticus* is genetically close to the other species—*S. suis*—which is known as a normal inhabitant of the oral cavity of pigs [67]. There are data that it is sometimes difficult to correctly identify *S. suis,* and even molecular methods, including PCR, have a risk of misidentifying *S. gallolyticus* as *S. suis* [68,69]. As in this study *S. suis* was detected in very low amounts, taxonomic distribution of *Streptococcus* species should be further studied for more accurate identification. The rest of the species in the colon were more equally distributed by their variety and numbers in both groups of pigs, although big differences were also observed regarding *Clostridium cellulovorans,* which had a higher prevalence in the control group, and different species of *Prevotella,* which were more prevalent in the experimental group. *C. cellulovorans* is known as widely distributed bacteria, which is also found in porcine respiratory tract; however, there is no clear information about its role in pigs.

Differences between microbial changes within the same animal group at the beginning and at the end of the experiment were recorded. While the main prevalent bacterial genera in both groups at the beginning of the experiment were *Prevotella*, *Clostridium*, *Barnesiella*, and *Streptococcus*, at the end of the experiment the most prevalent genera were *Clostridium*, *Escherichia*, *Lactobacillus*, and *Terrisporobacter* in the content of jejuni. In the large intestine, such differences were unequal: in caecum of the control group, *Prevotella* and *Barnesiella* predominated, whereas in the experimental group, the most prevalent were *Prevotella* and *Streptococcus*. In the colon, the most prevalent genera were *Streptococcus*, *Prevotella*, *Clostridium*, and *Barnesiella* in the control group and *Prevotella*, *Barnesiella*, and *Oligosphaera* in the experimental group. In spite of the fact that certain differences were determined between the groups at the end of the experiment, the same genera were detected in both groups only with different proportional count.

One of the most important questions raised in this study was to evaluate the influence of EEOs on the amount of probiotic bacteria in the GI tract of pigs. The data demonstrated that pigs receiving EEOs supplements had a higher amount of bacteria that are considered as probiotics, including *Lactobacillus*, *Bacillus*, *Bifidobacterium*, and *Escherichia*. Only *Streptococcus* and *Enterococcus,* which are also probiotic species, were more numerous in the control group of pigs. The overall number of probiotic bacteria in the experimental group was 1.2 times higher compared to controls and accounted for 27% of the total amount of bacterial reads in the gut of pigs. The number of *Lactobacillus* was 2.5 times higher, while the number of *Bifidobacterium* was 1.9 times higher in the experimental group. The amount of *Streptococcus*, however, was four times higher in the control group. As the lactic acid bacteria, including *Lactobacillus* and *Bifidobacterium,* are among the better described probiotic bacteria, the role of streptococci for the host is less investigated. This genus is mostly known as a pathogenic for pigs causing different diseases and, particularly, *S*. *suis* infections via skin injuries, which manifest as meningitis and other clinical expressions. There are data that natural probiotics can be a promising tool in cancer prevention and therapy for humans [70]; therefore, the obtained results can be important from the perspective of the usage of nutraceutical preparations containing EEOs. Moreover, *Lactobacillus* and *Bifidobacterium* are the genera already known as suppressors of cell proliferation in tumors; their action is associated with oxidative stress [70,71]. Although EOs anticancer potential is known by acting by multiple pathways and mechanisms, involving apoptosis, cell cycle arrest, antimetastatic and antiangiogenic action, increased levels of reactive oxygen and nitrogen species (ROS/RNS), and DNA repair modulation [72]-this study reveals that they can also act indirectly, by increasing populations of probiotic bacteria in the host. 

Our previous experiments with separate ingredients and intended for development of nutraceutical formulations have demonstrated good results in vitro, regarding antimicrobial activity of lactic acid bacteria, berries/fruit- by-products and EOs, which demonstrated bacteriostatic or bactericidal effect against wide spectrum of field pathogens [5,73,74]. Ultrasonication and fermentation of bovine colostrum allowed us to obtain decontaminated colostrum with increased functionality and reduced amount of cadaverine, histamine, and tyramine [74]. A new delivery system based on apple pomace-pectin gels ensured the viability of antimicrobial strains [75]. Current experiment in vivo demonstrated a positive effect of EEOs on the microbiota of the GI tract without reducing variety of species in the large intestine and increasing the number of probiotic bacteria. The study also demonstrated that EEOs combination has no cytotoxic effect at high concentrations (up to 50 µL/mL). The minimum inhibitory concentrations of EOs on different pathogenic strains in our previous study were lower [5] than concentrations which had no cytotoxic effect established in the present study. As there was a decrease of some probiotic bacteria, in particular, gram-positive cocci, more data are required about the possible action of the current EEOs combination on this group of bacteria and about the role of *Streptococcus* spp. in the gut of vertebrates. Moreover, as the EEOs had a certain negative impact on species variety in the ileum, studies on the role of minor species of bacteria in small intestine should be further studied. 

## 5. Conclusions

*Origanum vulgare* plant extract and *Mentha piperita* and *Thymus vulgaris* essential oils, alone and in combination, had no cytotoxic effect at concentrations up to 50 µL/mL after 48 h in MARC-145, PK-15, and Vero cell lines. The combination of these essential oils given to pigs orally in a form of tablet with bilayer coating, prepared by “liquid/solid” phase technology, resulted in the increase of probiotic bacteria in jejunum, caecum and colon using an experimental pig model.

## Figures and Tables

**Table 1:** Results of stability test (1 year) for tablets.

Samples	Thymol mg/mL	Carvacrol mg/mL	Menthol mg/mL
Tablets before (19 May 2019)	1.20206	0.0145364	0.56
Tablets after (19 May 2019)	1.02065	0.0112584	0.225
